# Microfluidic and Paper-Based Recombinase Polymerase Amplification Systems for Decentralized Diagnostics and Biosurveillance

**DOI:** 10.3390/mi17070825

**Published:** 2026-07-10

**Authors:** Hsing-Meng Wang, Sheng-Zhuo Lee, Lung-Ming Fu

**Affiliations:** Department of Engineering Science, National Cheng Kung University, Tainan 701, Taiwan; n98144028@gs.ncku.edu.tw (H.-M.W.); szlee0729@gmail.com (S.-Z.L.)

**Keywords:** recombinase polymerase amplification, microfluidic diagnostics, paper-based analytical devices, decentralized molecular testing, AI-assisted biosurveillance

## Abstract

Recombinase polymerase amplification (RPA) has become a central amplification strategy for decentralized molecular diagnostics because it operates rapidly at mild temperatures and requires far less thermal control than PCR. Its analytical value increases substantially when paired with microfluidic and paper-based platforms, where sample handling, reagent delivery, amplification, and signal readout can be organized within compact, low-power, and field-compatible formats. This review examines recent progress in microfluidic and paper-based RPA systems across biomedical diagnostics, food safety testing, environmental monitoring, and One Health biosurveillance. Particular attention is given to integrated device architectures, including centrifugal chips, capillary-driven platforms, microfluidic paper-based analysis devices (μPADs), electrochemical biosensors, CRISPR-assisted assays, digital microfluidic systems, and sample-to-answer cartridges. Biomedical applications now span respiratory viruses, reproductive and emerging infections, bacterial and parasitic diseases, pharmacogenomic markers, and cancer-related biomarkers. RPA-enabled platforms are moving steadily into food safety and environmental surveillance, covering pathogen detection, seafood and dairy monitoring, agricultural disease control, antimicrobial-resistance tracking, and airborne pathogen screening. At the same time, the field is shifting toward more intelligent diagnostic formats. Smartphone imaging, artificial intelligence (AI)-assisted interpretation, digital partitioning, cloud connectivity, and automated quality control are increasingly being built into rapid testing workflows, giving these systems greater portability, consistency, and decision-making value. Despite this progress, practical deployment still depends on robust sample preparation, multiplex stability, quantitative reliability, reagent storage, scalable fabrication, and regulatory validation. Continued convergence of RPA chemistry with microfluidics, paper devices, CRISPR recognition, electrochemical readout, and data-assisted interpretation is expected to support more robust and accessible molecular diagnostic workflows.

## 1. Introduction

Molecular diagnostics now occupies a central position in healthcare, food safety control, and environmental surveillance, largely because nucleic acid targets can be identified with high specificity and speed. Since the advent of the polymerase chain reaction (PCR), amplification-based assays have reshaped the detection of infectious agents, antimicrobial resistance markers, foodborne pathogens, and clinically relevant genetic signatures for precision medicine. Despite its central role in nucleic acid analysis, conventional PCR still relies on thermal cyclers, skilled operators, and laboratory-based workflows. Such dependence limits its use outside centralized facilities, particularly in resource-limited settings, point-of-care testing, and field surveillance, where timely decisions require diagnostic platforms that are compact, low-power, robust, and simple to operate [1,2,3,4,5,6].

PCR and recombinase polymerase amplification (RPA) occupy distinct positions in biomedical molecular testing. PCR remains the method most often chosen when clinical confirmation, quantitative measurement, and standardized laboratory reporting are required, largely because thermal cycling gives the reaction tight procedural control and a long record of validated performance. The same requirement for precise temperature cycling, specialized instruments, and laboratory operation also makes PCR less convenient for decentralized use. RPA addresses a different need. Amplifying nucleic acids at a mild, nearly constant temperature lowers the demand for thermal hardware and fits more readily into portable microfluidic chips, paper-based assays, and point-of-care devices. In practical terms, PCR is still the stronger option for laboratory confirmation and quantitative monitoring, whereas RPA is better matched to rapid screening, field testing, and biomedical diagnostics in settings with limited infrastructure. Table 1 summarizes these complementary roles, focusing on reaction conditions, instrument requirements, assay speed, and biomedical suitability.

Isothermal nucleic acid amplification has therefore gained considerable attention as a practical route beyond instrument-intensive PCR. Representative strategies include loop-mediated isothermal amplification (LAMP), nucleic acid sequence-based amplification (NASBA), helicase-dependent amplification (HDA), recombinase-aided amplification (RAA), and RPA [7,8,9,10]. Among these methods, RPA is particularly attractive because amplification proceeds efficiently at mild temperatures, typically 37–42 °C, without complex thermal control. Its short reaction time, modest hardware demand, and PCR-comparable analytical sensitivity make RPA well suited to decentralized molecular testing. Accordingly, RPA has become an important foundation for next-generation diagnostic systems designed for point-of-care and field-deployable applications [11,12].

### 1.1. Principle and Advantages of RPA

Unlike PCR, RPA does not depend on repeated heating and cooling to separate DNA strands. The reaction instead uses recombinase–primer complexes to probe double-stranded DNA for homologous sequences and to insert primers directly into the target region. The exposed single strand is protected by SSBs, while strand-displacing polymerase extends the invading primer from the opened duplex. At a constant temperature, repeated primer invasion followed by polymerase extension progressively enriches the target sequence, producing amplicons at levels suitable for detection [11,12].

RPA has become a favored choice in isothermal amplification because it brings together three practical qualities that are difficult to achieve in a single assay: speed, mild thermal operation, and compatibility with imperfect samples. In many reports, amplification is completed within 10–30 min, giving RPA a clear advantage when rapid molecular readout is required [11,12]. Its reaction temperature is low enough to ease power consumption and thermal-control demands, which is particularly useful for handheld, battery-powered, or field-deployable diagnostic devices. Equally important, RPA can maintain reliable amplification in the presence of inhibitors commonly carried over from clinical specimens, food-derived matrices, and environmental samples [13,14]. This balance of rapid amplification, simple heating, and matrix tolerance has made RPA especially well-suited to field molecular diagnostics, environmental surveillance, and home-based testing platforms [15,16].

Recent review articles further emphasize the expanding role of RPA in decentralized molecular testing. Analyses covering paper-based biosensors, nucleic acid point-of-care testing systems, and integrated microfluidic amplification platforms repeatedly identify RPA as a particularly suitable amplification chemistry for rapid, low-infrastructure diagnostics [16,17,18]. Its combination of speed, low thermal demand, and compatibility with portable formats has positioned RPA as a key amplification strategy for next-generation molecular diagnostic systems.

### 1.2. Integration of RPA with Microfluidic Technologies

While RPA simplifies nucleic acid amplification, practical deployment often requires integration with sample preparation, fluid handling, and signal detection systems. Microfluidic technology provides an ideal platform for achieving this integration because it enables precise manipulation of small fluid volumes, automated reagent delivery, reduced reagent consumption, and miniaturization of analytical workflows [19,20].

During the past decade, microfluidic implementation of RPA has expanded well beyond simple reaction chambers. Early platforms were shaped largely by continuous-flow microchannels and centrifugal disks, which provided controlled sample transport, reagent metering, and reaction timing in small-volume formats [21]. Device concepts later became more diverse and increasingly application-driven. Capillary-driven devices offered a practical way to move liquids without external pumps, thereby making RPA assays easier to operate in resource-limited settings [22]. Self-powered platforms made the device concept more practical for field testing, since fewer external components were required during operation [23]. In parallel, multiplex pathogen-detection chips broadened the analytical capacity of RPA, allowing several targets to be screened simultaneously within a single microfluidic layout [24,25]. More recent designs show a clear shift toward deployable diagnostic systems, particularly integrated respiratory-virus testing platforms [26] and automated sample-to-answer cartridges, in which sample processing, amplification, and signal readout are coordinated within one continuous workflow [27].

The pairing of microfluidics with RPA has changed the practical form of nucleic acid testing. Rather than treating extraction, amplification, and detection as separate laboratory steps, many recent devices place these functions inside a single disposable format, with fluid routing and reaction timing handled on-chip. This design logic has been discussed in several reviews of microfluidic nucleic acid testing, where RPA is frequently highlighted for lowering assay complexity and making molecular diagnostics more portable and accessible outside conventional laboratory settings [28,29]. CRISPR-integrated microfluidic systems have added a further layer of analytical capability. In these devices, microfluidic operation, isothermal amplification, and CRISPR-based recognition work together to improve specificity, support multiplex pathogen testing, and reduce operator intervention [30,31]. RPA is therefore no longer used only as a fast amplification step; in many recent devices, it now functions as part of a coordinated molecular testing workflow.

### 1.3. Emergence of Paper-Based RPA Diagnostics

Progress in microfluidic RPA diagnostics has been accompanied by a parallel shift toward paper-based analytical formats. Paper is not merely a passive support; its low cost, simple fabrication, biodegradability, scalable manufacture, and capillary-driven flow provide a practical basis for decentralized molecular testing [17,18]. These features are especially valuable when diagnostic systems must remain inexpensive, lightweight, and usable without pumps or complex peripheral equipment. Recent reviews have noted the increasing integration of nucleic acid amplification with paper-based biosensors [16]. RPA is especially compatible with this format because it proceeds rapidly under mild thermal conditions and requires little supporting instrumentation. Paper–RPA systems therefore provide a simple route to portable molecular testing, particularly for low-resource environments and home-based diagnostics [15].

Paper-based RPA has progressed from simple amplification–lateral-flow formats to more integrated diagnostic designs. Early platforms mainly coupled RPA with strip-based detection, offering a low-instrumentation route for molecular readout. Newer systems add functional modules that address sample preparation, fluid control, and matrix interference. This transition is evident in several recent design strategies. Some platforms introduce nucleic acid enrichment upstream of RPA to improve target availability before amplification [14]. Origami-inspired paper devices exploit folded geometries to control how liquids move across the substrate, allowing multistep reactions to be arranged in a compact format [32]. Vibration-assisted amplification [33] and inhibitor-removal components [34] address a different but equally important issue: maintaining reliable amplification when complex sample matrices interfere with enzyme activity.

Paper-based CRISPR–RPA devices have further expanded this platform by combining rapid amplification with sequence-specific CRISPR recognition. As a result, pathogen signals can be read visually with high sensitivity, while the assay remains largely free from complex instrumentation [35,36]. Together, these advances make paper-based RPA a practical route toward affordable molecular diagnostics for field use, low-resource settings, and home-based testing [16].

Table 2 sets representative paper-based RPA systems beside one another, comparing substrate choice, target analyte, readout strategy, assay duration, detection limit, and practical strengths. This side-by-side comparison highlights the diversity of paper-supported formats, ranging from lateral-flow strips and origami membranes to wax-gated μPADs, valve-integrated paper devices, and surface-engineered platforms. It also shows that the practical value of paper-based RPA extends beyond low-cost fabrication. In many cases, the substrate itself contributes to sample handling, reagent storage, capillary transport, reaction compartmentalization, or user-friendly signal readout.

### 1.4. Digital Microfluidics and Quantitative RPA Analysis

Conventional RPA is highly useful for qualitative detection, but obtaining a reliable quantitative readout is less direct. In a bulk reaction, amplification proceeds continuously, so the measured signal represents the collective reaction kinetics rather than discrete amplification events. Digital microfluidics offers a more countable format by splitting the sample into many isolated compartments, with each chamber operating as an independent amplification unit [44,45].

SlipChip technology offered one of the earliest demonstrations of compartmentalized microreactors for digital nucleic acid analysis [44]. Once the sample is distributed into discrete microreactors, positive and negative reaction chambers can be counted directly, allowing absolute nucleic acid quantification without relying on calibration curves. Later digital microfluidic systems carried this compartmentalized strategy further, using improved device layouts to handle more reactions in parallel, reduce manual operation, and make the workflow easier to automate [45].

Recent digital RPA systems have begun to combine automated droplet generation, compartment-based quantification, and high-throughput image analysis within a single analytical workflow [46,47]. By converting bulk amplification into many discrete reaction events, these platforms improve sensitivity for low-abundance targets and provide a clearer basis for quantitative readout. This digital format also fits naturally with automated image processing and machine-learning-assisted interpretation. Reaction images can be treated as structured datasets, allowing more consistent counting, classification, and quantitative biosensing than manual inspection alone [48,49]. In this way, digital RPA adds both analytical sensitivity and a data-driven layer to molecular diagnostic platforms.

### 1.5. Intelligent and Connected RPA Diagnostic Systems

Artificial intelligence (AI), smartphone imaging, cloud connectivity, and digital health infrastructure are starting to transform the design and interpretation of RPA diagnostics [15]. In several recent systems, smartphones are no longer used only as simple cameras; they support image acquisition, signal extraction, result interpretation, and data transmission to cloud-based platforms [50]. Deep-learning models have also been coupled with microfluidic diagnostic devices to reduce subjective readout differences and improve classification accuracy [51]. Automated digital microfluidics also shows a similar trend, with machine-learning tools being employed for droplet identification, signal quantification, and quality-control decisions [52,53].

AI-assisted molecular diagnostics are moving beyond single-test interpretation. When assay results pass from smartphones to cloud-linked health platforms, molecular testing begins to serve a wider surveillance role, supporting epidemiological monitoring, outbreak mapping, and population-scale disease assessment [54]. In this model, molecular tests no longer function only as isolated diagnostic events; they also become data-generating elements within healthcare and biosurveillance networks.

### 1.6. Scope of This Review

RPA diagnostics no longer belong to amplification chemistry alone. Microfluidic fluid control, paper-based device engineering, CRISPR-based target recognition, digital microfluidic partitioning, and AI-assisted signal interpretation have shaped their recent development. Each layer adds a different capability: simpler handling, lower-cost fabrication, higher specificity, quantitative readout, or more objective data analysis. As these elements come together, RPA-based platforms are finding roles not only in healthcare but also in food safety, environmental monitoring, and One Health surveillance [11,17,52]. Such breadth also creates a need for a more organized assessment of the field. The literature now spans conventional chips, paper devices, digital assays, CRISPR-coupled formats, smartphone readout, and AI-enabled analytical workflows. Microfluidic and paper-based RPA now reaches beyond benchtop amplification, taking shape as practical molecular assays for clinics, food-safety testing, environmental monitoring, and intelligent detection. This review maps that trajectory, then considers the stubborn gaps—sample handling, contamination, quantitative readout, scalable manufacture, commercialization, and wider use in decentralized diagnostics.

The adjacent domains raised by this comment were not overlooked; they were placed where their technical logic best fits the structure of the review. Veterinary testing, aquaculture surveillance, plant-pathogen screening, antimicrobial-resistance monitoring, and genetically modified crop detection are best placed within environmental and One Health biosurveillance, as these settings share the same field-facing pressures: irregular sampling conditions, matrix interference, biosafety concerns, and fragmented reporting routes [4,6,11,12,41]. Precision medicine, pharmacogenomic assays, mutation profiling, methylation analysis, and selected forensic applications fall more appropriately under biomedical diagnostics, where the analytical targets are mainly human-derived nucleic acids and performance evaluation is shaped by clinical validation requirements [8,9,10,13]. Biosecurity pathogen detection and outbreak preparedness also align with biosurveillance, particularly for portable RPA assays aimed at high-consequence or low-abundance targets [28,39,55]. Artificial intelligence, smartphone imaging, and cloud interpretation serve instead as enabling layers rather than separate biological domains [15,51,52,53,54]. This arrangement preserves a focused discussion around the most mature evidence for microfluidic and paper-based RPA systems while leaving room for adjacent fields as sample-to-answer platforms become stronger and more reliable.

## 2. Biomedical Applications

Biomedical diagnostics now demands molecular assays that can leave centralized laboratories without losing analytical reliability. This need is clear in infectious disease testing, antimicrobial resistance monitoring, cancer screening, and personalized medicine, where timely nucleic acid information can guide clinical action. Thermal cyclers, trained operation, and relatively long workflows continue to shape the use of PCR and RT-qPCR, which remain strong laboratory standards. Microfluidic and paper-based RPA systems offer a more practical route for near-patient testing, combining rapid amplification at mild temperatures with compact device formats and flexible signal readout [56,57]. The amplification step has also become the foundation for building versatility in the platform, incorporating CRISPR recognition, electrochemical sensing, digital microfluidics, smartphone imaging, and AI-assisted analysis. In this form, RPA works less as a single reaction and more as a diagnostic engine for clinical screening, infectious disease detection, biomarker analysis, and personalized medicine.

### 2.1. Respiratory Viral Infections

Microfluidic RPA provides a practical route for respiratory-virus diagnostics by combining rapid amplification, simplified handling, and compact chip-based readout. Recent platforms have supported SARS-CoV-2 aerosol monitoring; multiplex detection of SARS-CoV-2, RSV, influenza viruses, and parainfluenza viruses; and influenza subtyping from respiratory samples [19,37,56,57,58]. Reported assays typically finish within 10–45 min, with sensitivities around 10–20 copies/µL. This performance makes microfluidic RPA useful not only for decentralized diagnosis but also for environmental monitoring and faster recognition of respiratory viral coinfections.

In the work of Li et al. [55], SARS-CoV-2 aerosols were monitored using a microfluidic RPA cartridge that brought together cyclone-based air sampling, chitosan-assisted RNA enrichment, and in situ tetra-primer amplification. The same cartridge was applied to aerosols, nasopharyngeal swabs, saliva, exhaled breath condensates, and air-outlet swabs. Fluorescence signals became readable within 25 min, and the detection limit reached 20 copies/mL. Its higher positivity in low-biomass and warm aerosol samples indicates value for noninvasive screening and environmental early warning.

For multiplex respiratory diagnosis, Liu et al. [26] developed MAPnavi, a sealed microfluidic platform that links nested RPA with CRISPR/Cas12a fluorescence readout. Within 40 min, the assay detected SARS-CoV-2 targets, RSV, influenza A/B, 2009 H1N1, H3N2, and an internal control. Analytical sensitivity reached 1 copy/µL, and testing of 171 nasopharyngeal samples showed strong agreement with reference methods. MAPnavi therefore extends microfluidic RPA beyond single-virus confirmation, offering a closed and contamination-controlled format for syndromic respiratory surveillance.

**Figure 1:**
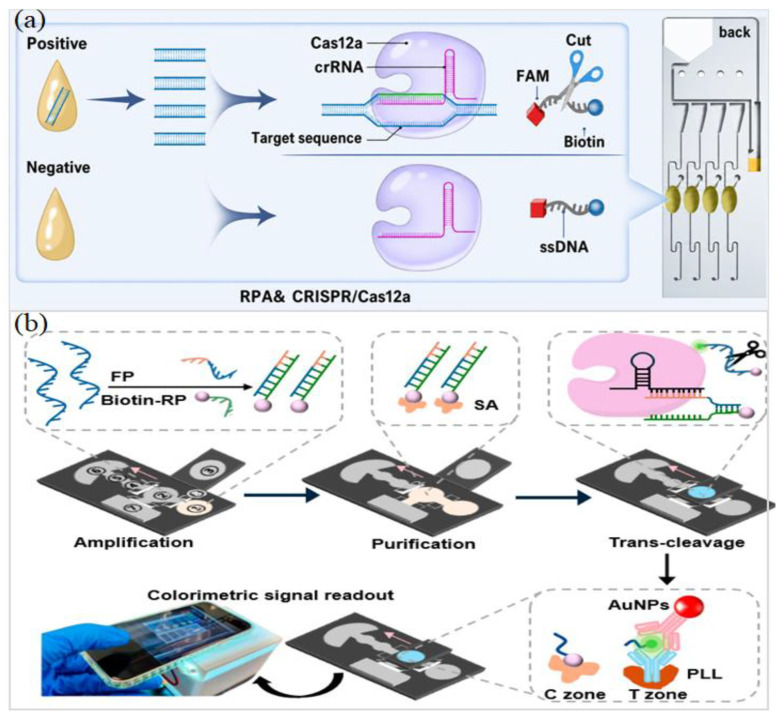
(**a**) Schematic illustration of a CRISPR-assisted microfluidic RPA chip for the detection of multiple respiratory viruses. (Reprinted from Ref. [59]). (**b**) Schematic representation of a μPAD-based platform integrating RPA and colorimetric CRISPR/Cas12a for HPV16 E7 detection. (Reprinted from [36]).

Li et al. [59] described a passively driven RT-RPA-CRISPR/Cas12a chip for respiratory virus screening in infrastructure-limited settings. The assay detected influenza A/B, human parainfluenza virus, and SARS-CoV-2, as shown in Figure 1a. Its workflow remained deliberately simple: 10 min for sample preparation, 35 min for the chip reaction, and colloidal-gold lateral-flow readout after RT-RPA–Cas12a recognition. Without fluorescence optics, the assay detected targets at approximately 10 copies/µL and achieved clinical sensitivity and specificity of 98.44% and 100%, respectively. Capillary/gravity-driven transport and lyophilized on-chip reagents further reduced dependence on pumps, optical readers, and skilled operation.

Lu et al. [60] demonstrated rapid influenza subtyping using a PMMA centrifugal RPA chip covering H1N1, H2N2, H3N2, H5N1, H7N9, and influenza B. The assay used extracted respiratory RNA, operated at 42 °C, and produced results within 10 min. Sensitivity was retained at low viral copy numbers, and results from all 180 clinical samples matched those obtained by RT-PCR. Collectively, these studies show that microfluidic RPA is no longer limited to rapid amplification chemistry; it is becoming a complete diagnostic architecture for respiratory-virus screening, subtyping, and surveillance.

### 2.2. Reproductive, Blood-Borne, and Emerging Viral Infections

RPA-enabled microfluidic and paper-based platforms are reshaping diagnostics for reproductive, blood-borne, and emerging viruses by coupling mild isothermal amplification to diverse, field-compatible readouts, including CRISPR, electrochemical, colorimetric, lateral-flow, and droplet formats. Across HPV, sexually transmitted pathogens, MPXV, WNV, and hCMV, these systems shorten workflows, reduce equipment dependence, and improve access to decentralized testing in real-world settings [61,62,63,64,65].

Xu et al. [66] described a cervical-swab workflow that integrates multiplex RPA, Cas12a reporting, and microfluidic spatial coding to distinguish nine HPV genotypes covered by the 9-valent vaccine. Genotype assignment relies on position rather than multiple dyes: a single fluorophore is used while reactions are distributed across a 30-well starburst chip, completing subtyping in approximately 40 min. After amplification, signals extended from blank to 1 × 10^−16^ M, with a detection limit of 2.67 × 10^−19^ M (0.26 aM). In a 100-specimen clinical evaluation, sensitivity reached 97.8% and specificity 98.1%.

For HPV16/18 genotyping, Zhao et al. [67] used a dual-droplet CRISPR/Cas12a microdevice in which green and red droplets served as separate reaction compartments for target discrimination. The assay reached approximately 10^−18^ M for HPV16, corresponding to about one copy per reaction, with a turnaround time of approximately 30 min. Across 20 clinical samples, sensitivity was 92.3%, and specificity was 100%. Physical compartmentalization suppresses crosstalk while preserving multiplex capability, making the format well suited to compact HPV genotyping.

Complementary HPV implementations further illustrate the breadth of RPA-enabled formats. Zhang et al. [36] designed a μPAD for HPV16 E7 dsDNA that integrates lyophilized RPA/CRISPR reagents with AuNP–anti-FAM colorimetry for naked-eye readout, achieving 100 pM detection within 60 min, as shown in Figure 1b. Nakowong et al. [68] paired RPA with a methylene-blue electrochemical genosensor to detect HPV16 in cervical tissue samples; the assay read down to 0.23 copies/μL and matched PCR across 40 specimens.

Sexually transmitted and emerging viral infections have also benefited from streamlined RPA workflows. A hand-warmer-heated paper device reported by Chu et al. [38] integrates nucleic-acid isolation, RPA, and lateral-flow readout for Neisseria gonorrhoeae in urine. The assay was completed in 30–35 min and detected 10 DNA copies or 1 CFU/mL, improving field accessibility relative to tube-based RPA and PCR. Hwang et al. [69] established RT-RPA–Cas12a DETECTR formats for West Nile virus lineages 1a and 2, with 10^2^ RNA copies for the two-step and filter-assisted formats and 10^3^ copies for the one-step format. Together, these examples show that the clinical value of RPA is greatest when amplification is embedded in devices that manage transport, partition reactions, stabilize reagents, and translate recognition into readable optical, electrochemical, or lateral-flow signals.

### 2.3. Tuberculosis, Bacterial, and Parasitic Infectious Diseases

Microfluidic RPA systems now extend from tuberculosis (TB) and bacterial infection testing to malaria genotyping [70,71]. Their practical value lies in short assay times, low-temperature amplification, compact fluid handling, and visually or digitally readable signals that reduce dependence on culture, microscopy, and centralized PCR. Saikaew et al. [72] introduced TB-GoldDx as a visual tuberculosis assay built on IS6110-specific RPA and unmodified AuNP colorimetry. Clinical-isolate and sputum-derived DNA provide a more clinically relevant benchmark for tuberculosis screening than template-only experiments. After amplification at 39 °C for 20 min, salt-induced AuNP aggregation produced a distinct color change, with a detection limit of 0.001 ng H37Rv DNA (≈210 bacilli per 25 µL reaction). Across the 100-strain set, sensitivity was 95.83%, and specificity remained at 100%. In sputum, sensitivity was 81.43% overall, rising to 95.45% in AFB 3+ specimens. This low-instrument format is well suited to screening, whereas low-burden sputum remains best interpreted alongside confirmatory testing.

For broad bacterial diagnosis, Xie et al. [73] reported MiND-DMF, a digital microfluidic platform integrating DNA extraction, RPA, and CRISPR detection for *P. aeruginosa*, *H. influenzae*, *K. pneumoniae*, *E. coli*, *S. aureus*, and MRSA. A broad spectrum of clinical matrices—including blood, urine, bronchoalveolar lavage fluid, nasal secretions, appendiceal pus, and otorrhea—was compatible with the chip. Run at 37 °C, the workflow finished in ≤55 min and reached an LOD of 100 CFU/mL; sensitivity was 100% and specificity 98–100% in 50 clinical samples. A key strength is the shift from manual microbiology toward an automated, sample-to-answer identification workflow, which streamlines handling and can inform earlier, evidence-based antibiotic selection.

For urinary tract infection (UTI)-associated bacteria, Chen et al. [74] described an autosampling microfluidic assay for five major pathogens (*E. coli*, *K. pneumoniae*, *A. baumannii*, *E. faecium*, and *E. faecalis*). Syringe filtration was used to concentrate urine and related clinical fluids; rapid lysis followed, and the lysate was subsequently vacuum-partitioned inside a PDMS chip. Analytical sensitivity reached 5 DNA copies per reaction and 10^2^ CFU/mL in urine; the complete workflow closed in 55 min. In a 299-specimen study, agreement with culture corresponded to 91.1% sensitivity and 100% specificity. Xiu et al. [75] illustrated parasitic diagnostics by coupling dried-blood-spot DNA with a microfluidic RPA–CRISPR readout for malaria screening and Plasmodium genotyping. Five species were resolved with universal primers and species-specific crRNAs. The two-step format detected 10 copies per reaction, whereas the one-pot chip achieved ~10^2^ copies/µL. Clinical testing reported 98.41% sensitivity and 92.86% specificity, with 90.91% concordance relative to PCR sequencing.

### 2.4. Precision Medicine and Cancer Biomarkers

RPA workflows designed for precision diagnostics now do more than amplify a target and report an endpoint signal. Genotypes, mutations, and methylation states can be read from buccal swabs and tumor-derived DNA using fluorescence, lateral-flow, electrochemical, or CRISPR outputs, enabling faster, more portable alternatives to sequencing, PCR, and bisulfite-dependent assays [76,77,78,79].

**Figure 2:**
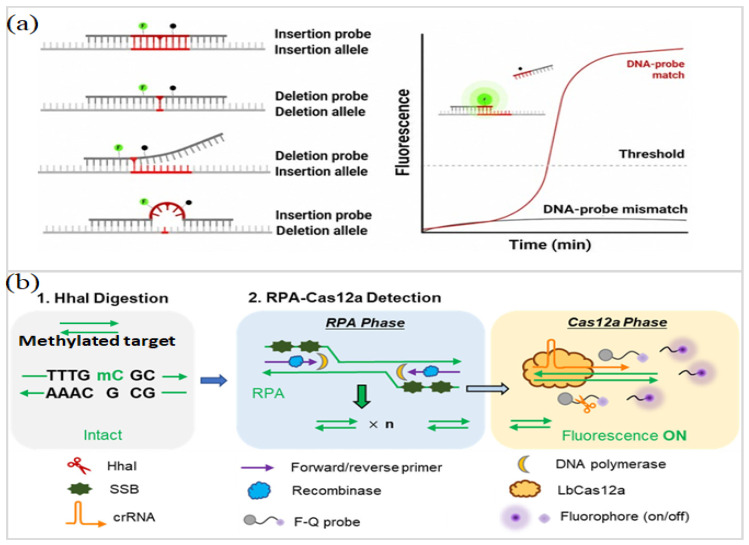
(**a**) Schematic of allele-specific RPA-exo probe design for insertion–deletion genotyping and fluorescence-based discrimination of matched and mismatched DNA–probe hybrids. (Reprinted from [80]). (**b**) Schematic illustration of a methylation-sensitive restriction endonuclease-assisted RPA–CRISPR/Cas12a method for methylation detection. (Reprinted from [83]).

De Keyzer et al. [80] reported a real-time RPA–exo probe fluorescence assay for insertion–deletion genotyping using reference DNA and purified blood-derived DNA, as shown in Figure 2a. The reaction proceeded isothermally at 39°C and generated genotypes within 20 min, reaching 97.07% accuracy with 1 ng input, reliable profiling at 250 pg, and partial recovery at 31 pg. By replacing thermal cycling and electrophoretic separation with low-temperature probe readout, this format provides a credible path toward portable genotyping beyond PCR–capillary electrophoresis and massively parallel sequencing.

In a related study, Pang et al. [81] processed buccal-swab lysates with a smartphone-controlled duplex RPA–LFD assay strengthened by locked nucleic acid probes. The readout was completed within 25 min, and both HLA-B*58:01 and β-globin were detected at 10 copies/µL. Across 15 clinical samples, the assay showed full concordance with PCR-SBT. The two studies together point to a clear trend toward portable HLA screening before prescribing allopurinol, with internal controls helping to reduce false-negative calls and improve pharmacogenomic decision-making.

Thoeny et al. [82] interrogated three PIK3CA hotspot mutations by coupling bio-tin-dCTP–labeled RPA to a screen-printed gold-electrode array with electrochemical immunoreadout. RPA alone reached a detection limit of 100 copies/µL; after electrochemical transduction, the thresholds shifted to 229 copies/µL with asymmetric ssDNA and 224 copies/µL with λ-exonuclease–derived ssDNA. Wild-type blocking oligonucleotides gave the assay a cleaner separation between mutant and wild-type alleles, allowing reliable genotype assignment when the mutant fraction was above approximately 20%. Evidence from plasma ctDNA and primary tumor specimens is still scarce, leaving clinical validation as an important next step.

For epigenetic screening, Lu et al. [83] reported an HhaI-assisted glycerol-enhanced RPA–CRISPR/Cas12a assay for a CNE2-specific methylation locus associated with nasopharyngeal carcinoma, as shown in Figure 2b. HhaI digestion removed unmethylated DNA, leaving methylated targets intact for RPA–Cas12a fluorescence detection. The method achieved a detection limit of 100 aM, a linear range of 100 aM–100 fM, and methylation-level discrimination down to 0.1%. Cell-line testing separated CNE2 from NP69, although clinical validation remains essential.

Overall, these studies position RPA as a practical engine for decentralized precision diagnostics. Its strongest role is not to replace sequencing or PCR in all settings but to bring selected genotyping, mutation, and methylation tests closer to prescribing decisions, early screening, and resource-limited clinical workflows. Table 3 collates representative RPA-enabled platforms, organized by target, amplification conditions, sensing chemistry, readout modality, and analytical performance. The side-by-side view illustrates how integrated modules—such as CRISPR, electrochemical transduction, or multiplexed designs—shorten workflows while retaining clinically meaningful sensitivity.

### 2.5. Challenges and Prospects in Biomedical RPA Diagnostics

Biomedical RPA diagnostics are moving beyond “fast amplification” toward sealed, automated formats that deliver interpretation-ready outputs. Among current directions, CRISPR-coupled RPA is especially consequential: Cas12a/Cas13a recognition adds sequence-level verification to low-temperature amplification, tightening specificity for multiplex respiratory panels, HPV subtyping, and bacterial pathogen detection [26,66,84,85,86,87]. Spatially encoded microfluidic designs extend this logic by partitioning targets into predefined reaction addresses, enabling high-plex identification with straightforward optical readout rather than increasingly complex dye and probe combinations [66].

Decentralized formats are gaining momentum. Smartphone-controlled disks, hand-driven centrifugal chips, paper-based assays, and AI-assisted image decoding collectively reduce reliance on centralized laboratories [51,81]. R-CHIP is a notable example of this change because it combines RPA, CRISPR readout, manual actuation, smartphone imaging, and ResNet-based classification for high-risk HPV screening [51]. In parallel, LNA-enhanced duplex RPA for HLA-B*58:01 extends the approach toward pharmacogenomic decision support [81], while self-driven 3D-printed chips with glucose-meter readout suggest an inexpensive route to outbreak-site testing [88].

Because clinical matrices contain inhibitors, variable viscosity, low target abundance, and background signals, sample preparation continues to dictate assay performance and remains a persistent vulnerability in sample-to-answer systems [84,86]. Multiplex RPA hinges on disciplined primer stoichiometry and sequence-level vetting, since even minor imbalances can seed primer-dimer artifacts, promote cross-reactivity, and elevate mismatch-driven false positives [66,81]. The most translatable systems will combine robust front-end extraction, lyophilized shelf-stable reagents, sealed cartridges, reliable valving, controlled heating, automated quality checks, interpretation-ready analytics, and secure data transfer into clinical reporting workflows [51,84,87,88].

## 3. Food Safety Applications

Food safety sits at the intersection of public health and supply-chain resilience, with hundreds of millions of illnesses each year and costs that ripple across production, processing, and distribution. Hazards are diverse—foodborne bacteria and viruses, spoilage consortia, antimicrobial-resistant organisms, and deliberate adulteration—so rapid checks near the point of risk are essential. Routine surveillance, however, still relies heavily on culture, ELISA, PCR, and sequencing, which typically demand laboratory infrastructure, specialized personnel, and multi-hour timelines [11,89]. Microfluidic and paper-based RPA platforms narrow this gap: mild-temperature isothermal amplification pairs naturally with miniaturized fluid handling and portable readouts, bringing molecular verification into field and factory settings. Recent systems further integrate CRISPR confirmation, smartphone imaging, electrochemical transduction, digital microfluidics, and automated front-end preparation, broadening both throughput and target scope for foodborne surveillance [12,35,90].

### 3.1. Detection of Foodborne Bacterial Pathogens

Culture-first confirmation is steadily giving way to RPA-enabled devices that unify sample handling, amplification, and portable readout within a single workflow. Recent polymeric, electrochemical, and microfluidic formats compress time-to-answer from days to minutes while maintaining sensitivity in matrix-rich foods, including poultry, leafy vegetables, and milk [90,91].

**Figure 3:**
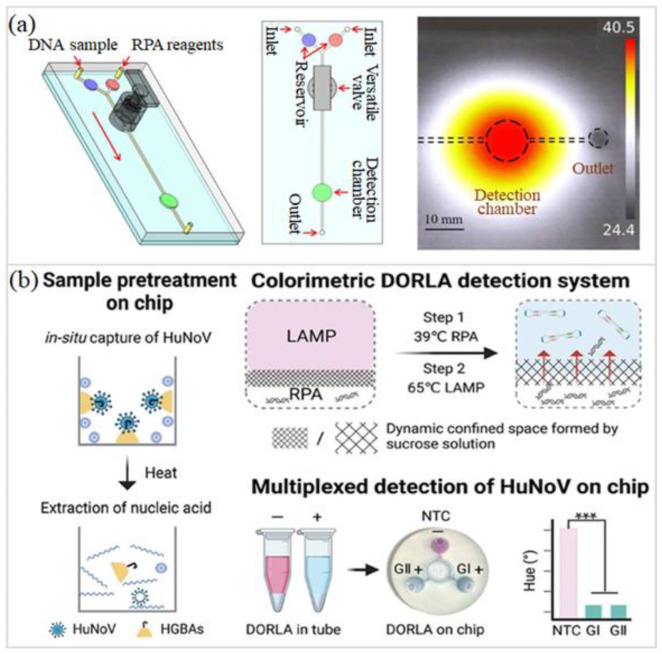
(**a**) Schematic illustration of the microfluidic RPA chip for Salmonella detection and thermal infrared image of the detection chamber. (Reprinted from [50]). (**b**) Schematic representation of the fully integrated microfluidic platform for multiplexed detection of HuNoV GI and GII in fecal samples, based on the dynamic confined-space one-pot RPA–LAMP system (DORLA), *** means *p* < 0.001 (n = 3). (Reprinted from [97]).

Chen et al. [92] demonstrated a hybrid paper–polymer microfluidic assay for Campylobacter jejuni in which DNA is captured by a dipstick, amplified by RPA for 20 min, and read on a lateral-flow strip. Using the poultry-associated hipO locus as the marker, the assay achieved limits of 46 CFU/mL after cellulose-paper extraction and 460 CFU/mL in the fully integrated format. In chicken meat, 10^1^–10^2^ CFU/g required 5–10 h enrichment, whereas loads above 10^2^ CFU/g were verified directly, compressing culture confirmation (~6–8 days) into a field-ready workflow. Chen et al. [32] translated Salmonella enterica detection into a polyethersulfone (PES)-based origami microfluidic format, in which dipstick nucleic-acid extraction feeds directly into RPA and lateral-flow visualization. A visible readout was obtained within 20 min, with 100% specificity and a detection limit of 260 CFU/mL. After 6 h of enrichment culture, a contamination level of 58 CFU/mL was detected in lettuce, chicken breast, and milk. The choice of PES was central: compared with chromatography paper, it better preserved amplification efficiency and thereby supported a more sensitive sample-to-answer workflow. Complementarily, Jin et al. [50] introduced a PDMS microfluidic biosensing system with an integrated valve for mixing and flow control, coupled to real-time smartphone fluorescence detection of *S. Typhimurium* (as shown in Figure 3a); the assay reached 1.0 × 10^2^ copies μL^−1^ within 30 min, approaching qPCR-level sensitivity while avoiding thermal cycling and bulky optics.

Electrochemical CRISPR–RPA formats are increasingly being adapted for quantitative monitoring of foodborne bacteria. Guo et al. [93] constructed an MWCNTs–Au-modified screen-printed carbon electrode for S. aureus quantification. In culture medium, the sensor showed a linear response over 1.04 × 10^1^–1.04 × 10^8^ CFU/mL and reached a detection limit of 3 CFU/mL. The tests in milk further confirmed matrix applicability across 1.07 × 10^1^–1.07 × 10^7^ CFU/mL. Guo et al. [94] further refined this strategy using MoS_2_–Au electrodes, retaining the 3 CFU/mL detection limit while extending the working range to 1.06 × 10^1^–1.06 × 10^8^ CFU/mL, together with strong selectivity in milk. Chen et al. [95] subsequently introduced a one-pot RPA–CRISPR/Cas12a electrochemical lateral-flow strip for Salmonella, reaching 3.84 CFU/mL by electrochemical readout and 384 CFU/mL by visual inspection, while limiting aerosol contamination associated with tube opening. Taken together, these studies show that foodborne bacterial RPA diagnostics are shifting toward closed, matrix-tolerant, and user-readable formats. The next critical step is multicenter validation using naturally contaminated foods and harmonized quantitative thresholds.

### 3.2. Viral and Multiplex Detection of Foodborne Pathogens

Food-safety diagnostics based on RPA are gradually extending beyond single-bacterium assays toward viral monitoring and multiplex pathogen screening [35,96]. Compact analytical formats that combine microfluidics or paper substrates with CRISPR, LAMP, SERS, electrochemical transduction, and smartphone-assisted interpretation are shaping this shift. Such integration gives the assay a more field-oriented character: shorter turnaround, lighter instrumentation, and less reliance on centralized PCR facilities or culture-based confirmation.

For viral diagnostics, Chen et al. [97] developed a fully integrated microfluidic RPA–LAMP system for simultaneous human norovirus (HuNoV) GI and GII detection in fecal samples, as shown in Figure 3b. The design brought histo-blood group antigen (HBGA)-based viral capture, nucleic-acid extraction, and sucrose-mediated confinement into the same chip before visual reporting. A staged reaction with RPA at 39 °C and then LAMP at 65 °C kept amplification sealed and minimized carryover. The assay resolved GI and GII at 10 and 1 copy/μL, respectively, and produced clinical readouts that tracked closely with RT-qPCR. Yin et al. [39] adapted norovirus detection to a lyophilized paper-based RT-RPA–CRISPR/Cas12a assay. The system was validated with tap water, lettuce, and oyster matrices over a range of 10^0^–10^8^ copies/µL with a detection limit of 1 copy/µL. Smartphone-readable fluorescence signals were obtained within 45 min, and assay activity was preserved after 8 weeks of room-temperature storage. The method also detected six oyster samples that had been confirmed positive by RT-qPCR. This format is especially attractive for field surveillance, where cold-chain storage, benchtop optical readers, and experienced personnel are often unavailable.

Multiplex bacterial detection has advanced in parallel. Shang et al. [98] broadened multiplex RPA diagnostics with an integrated, smartphone-assisted micro-platform targeting eight foodborne bacteria: *Salmonella* Typhimurium, *S. aureus*, *B. cereus*, *P. aeruginosa*, *E. coli* O157, *C. sakazakii*, *L. monocytogenes*, and *V. parahaemolyticus*. In this microfluidic platform, bacterial DNA was captured on a membrane and then amplified with preloaded, freeze-dried real-time RPA reagents, while smartphone fluorescence imaging served as the readout; the full workflow was completed within 60 min. Culture assays showed limits of 10^3^ CFU/mL for six targets, whereas *B. cereus* and *C. sakazakii* required 10^4^ CFU/mL. The method remained applicable to water, milk, fish, and chicken samples, although matrix interference shifted some detection limits upward. Han et al. [99] addressed a practical barrier in on-site testing by introducing a finger-actuated modular pressurizing-pump microfluidic device for milk analysis. The device detected *E. coli* O157, *Salmonella* spp., and *L. monocytogenes* with limits approaching 1 CFU/mL. These studies move food-safety testing closer to field use, with portable and multiplexed readouts replacing laboratory confirmation. Broader adoption is now limited less by analytical sensitivity than by sample cleanup, matrix tolerance, and validation using naturally contaminated foods.

### 3.3. Aquatic and Dairy Product Safety Monitoring

RPA is redefining aquatic and dairy safety monitoring by moving rapid molecular testing into complex food matrices. Coupled with microfluidics, μPADs, CRISPR/Cas12a, electrochemical sensing, smartphone fluorescence, and test strips, these assays now support seafood pathogen screening, species authentication, and milk-quality surveillance [93,99,100].

For seafood-associated Vibrio parahaemolyticus, Fang et al. [101] developed a centrifugal microfluidic biosensor that coupled one-pot RPA–CRISPR/Cas12a amplification with in-run standard-curve calibration. Using shrimp, mackerel, oyster, and field-collected shrimp samples as target matrices, the system achieved a detection limit of 6.08 copies/μL, a 10^0^–10^4^ copies/μL quantitative range, and qPCR-comparable performance while shortening quantitative analysis from culture-scale timelines to approximately 1.5 h. Chen et al. [40] introduced a foldable μPAD for rapid Vibrio parahaemolyticus screening in seawater and seafood, using micro-vibration-assisted mixing to improve assay performance. Using air-dried RPA reagents together with 40 °C amplification and smartphone fluorescence imaging, the device achieved 10^2^ CFU/mL in 20 min. Results from 36 seafood samples purchased from local markets closely paralleled those from routine culture analysis.

**Figure 4:**
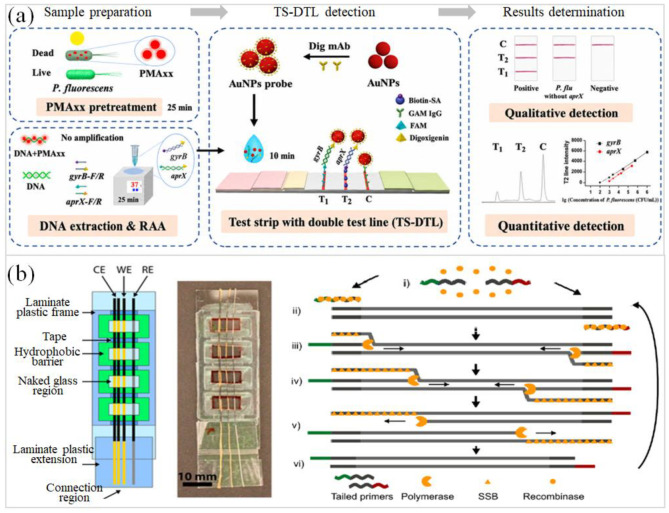
(**a**) Schematic illustration of recombinase-aided amplification coupled with a dual-test-line strip for *P. fluorescens* detection. (Reprinted from [103]). (**b**) Schematic diagram of gold-coated thread-based multi-target electrochemical biosensors for Ostreopsis cf. ovata and Ostreopsis cf. siamensis detection and the tailed-primer RPA (i–vi) cycle. (Reprinted from [104]).

Seafood authentication shows how microfluidic RPA can move beyond pathogen screening. Xu et al. [102] applied a real-time eight-sample chip to 141 commercial seafood products, targeting Atlantic cod, sablefish, and toothfish. The chip processed eight samples in parallel, completed RPA within 20 min, reached 10 copies/μL plasmid or 10^3^ fg/μL genomic DNA, and exposed a 31.91% label–identity mismatch. In dairy monitoring, Guo et al. [94] described electrochemical RPA–CRISPR/Cas12a biosensors for Staphylococcus aureus analysis in milk, using either MWCNTs–Au or MoS_2_–Au electrode interfaces to convert Cas12a-mediated reporter cleavage into a decrease in DPV current. Both systems reached a detection limit of 3 CFU/mL and produced linear responses across 10^1^–10^8^ CFU/mL-scale bacterial concentrations. With the modified electrodes, milk-derived background effects were restrained, and bacterial quantification remained more stable. Zhang et al. [103] described milk spoilage monitoring with a dual-test-line strip based on recombinase-aided amplification (RAA) for viable Pseudomonas fluorescens targeting gyrB and aprX, as shown in Figure 4a. Naked-eye detection reached 50 and 250 CFU/mL, while quantitative limits in milk were 37 and 233 CFU/mL, respectively; results from spiked milk showed full agreement with culture. Overall, the main bottleneck now lies in cleanup, reagent stability, matrix tolerance, and validation in naturally contaminated aquatic and dairy products.

### 3.4. Electrochemical Food Safety Biosensors

Electrochemical signal measurement helps move RPA-based food safety assays from instrument-dependent analysis toward portable testing with quantitative output. These platforms convert nucleic-acid recognition into measurable current responses instead of using fluorescence imaging, gel electrophoresis, or centralized PCR systems [93,94]. Recent studies have paired RPA with CRISPR/Cas chemistry, nanostructured electrodes, paper or thread supports, and microfluidic formats, enabling rapid analysis of bacterial pathogens, seafood hazards, toxic microalgae, and GMO markers. The same concept is particularly valuable for genetically modified organism (GMO) traceability, where isothermal amplification and nanomaterial-modified transducers can support sensitive screening directly at the point of need [89,95].

Electrochemical coupling brings a practical readout to aquatic food surveillance. In Xu et al. [102], fish-associated Vibrio parahaemolyticus was detected using an RPA-mediated E-CRISPR biosensor built around the tdh and trh loci. RPA first enriched the target sequences; target-activated Cas12a then cleaved ssDNA reporters fixed on the electrode, translating sequence recognition into an electrochemical current change. The assay produced a linear response over 10^1^–10^6^ CFU/mL and reached a detection limit of 32 CFU/mL. Hanze et al. [104] employed gold-coated thread electrodes to read RPA-amplified toxic microalgal DNA electrochemically, as shown in Figure 4b. More than a sensing interface, this roll-to-roll-compatible design points to a scalable route for multi-target surveillance in aquatic environments. Dong et al. [105] advanced electrochemical CRISPR detection with an immobilization-free Cas13a format. Following RPA preamplification, the assay reached 30 zM sensitivity; in a multi-chamber chip, it resolved six pathogens within 50 min. Overall, electrochemical RPA biosensors are shifting toward compact, multiplex, matrix-aware formats; remaining hurdles lie in electrode reproducibility, simpler sample cleanup, and validation with naturally contaminated foods. Table 4 compares representative RPA-based food safety biosensors, emphasizing sensing chemistry, readout strategy, analytical range, and detection limits.

### 3.5. Challenges and Future Perspectives in Food Safety Testing

Microfluidic RPA systems have advanced rapidly, but their use in food safety testing still faces several barriers before routine deployment becomes realistic. Among them, sample complexity remains one of the most persistent obstacles. Food matrices are inherently heterogeneous, often containing fats, proteins, polysaccharides, salts, pigments, and other endogenous inhibitors that can compromise nucleic-acid recovery, disrupt microfluidic transport, or reduce amplification efficiency [11,12]. Reliable field use therefore calls for pretreatment modules that are both simple and matrix-aware. Filtration, concentration, inhibitor removal, nucleic-acid capture, and reagent-compatible extraction should be built into a single disposable workflow rather than treated as separate preparation steps. A second limitation is quantification. Many RPA platforms still provide endpoint color or fluorescence signals, which are useful for screening but less suitable for risk assessment, regulatory thresholds, or contamination-level tracking. Digital microfluidics, real-time fluorescence, and electrochemical transduction may help close this gap by improving signal normalization, dynamic range, and user-independent interpretation [19,89]. Third, multiplex detection must move beyond placing several reactions side by side. Reducing cross-reactivity and false-positive signals will require improved primer design, CRISPR-assisted target discrimination, compartmentalized reactions, and automated fluidic control [35,50,95]. In general, microfluidic and paper-based RPA technologies are already faster, easier to use, and more portable than culture, PCR, and ELISA. Their broader adoption will depend on sealed cartridges, lyophilized reagents, smartphone- or AI-assisted readout, scalable fabrication, and validation using naturally contaminated foods.

## 4. Environmental Monitoring Applications

Environmental monitoring is now a crucial aspect of public health, agricultural protection, ecosystem stewardship, and early warning against emerging infections. Air, water, soil, crops, wildlife, and aquaculture environments can all act as reservoirs or transmission routes for pathogenic microorganisms. Conventional surveillance still depends largely on culture, PCR, or sequencing, approaches that remain accurate but slow, instrument-intensive, and tied to centralized laboratories; such constraints can delay intervention during outbreaks or contamination events [11,12]. Microfluidic and paper-based RPA platforms offer a more deployable alternative. Coupling RPA with compact fluidic layouts brings molecular testing directly to the sampling site, allowing rapid screening of environmental pathogens, crop-associated diseases, antimicrobial resistance genes, and zoonotic agents without extensive laboratory infrastructure [106,107]. Selectivity, operational control, and field reliability can be improved when RPA is coupled with CRISPR verification, electrochemical readout, or cartridge-level workflow integration.

### 4.1. Agricultural and Aquatic Environmental Biosurveillance

Agricultural and aquatic biosurveillance has shifted from laboratory-bound confirmation toward portable RPA-enabled systems. Recent microfluidic and paper-based platforms support airborne spore monitoring, forestry pathogen detection, shrimp disease surveillance, and aquatic pathogen quantification, offering faster operation, lower infrastructure demand, and stronger field relevance [11,108].

Yang et al. [106] used airborne spores as early sampling targets and incorporated this strategy into a microfluidic nucleic acid detection chip for rice smut surveillance. The platform links spore capture, on-chip germination, RPA, and lateral-flow dipstick detection into a compact testing route. A key step is the short germination stage: once the spores develop into delicate mycelial structures, DNA release becomes possible without liquid-nitrogen grinding or hands-on extraction. Within 1 × 10^2^–1 × 10^5^ CFU/mL, the assay generated a visual sample-in–answer-out response, making it a practical field-oriented option compared with microscopy, staining, or PCR-based laboratory analysis. For field forest protection, Sun et al. [109] developed a portable microfluidic device for detecting pinewood nematodes in pine dust. The system combines boiling pyrolysis, RPA amplification, fluid metering, mixing, temperature control, and gold immunochromatographic strip detection and can obtain visual results within 40 min. Its main value is rapid field warning, not wide-range quantification, as it avoids Baermann extraction and morphology-based identification in routine nematode testing.

**Figure 5:**
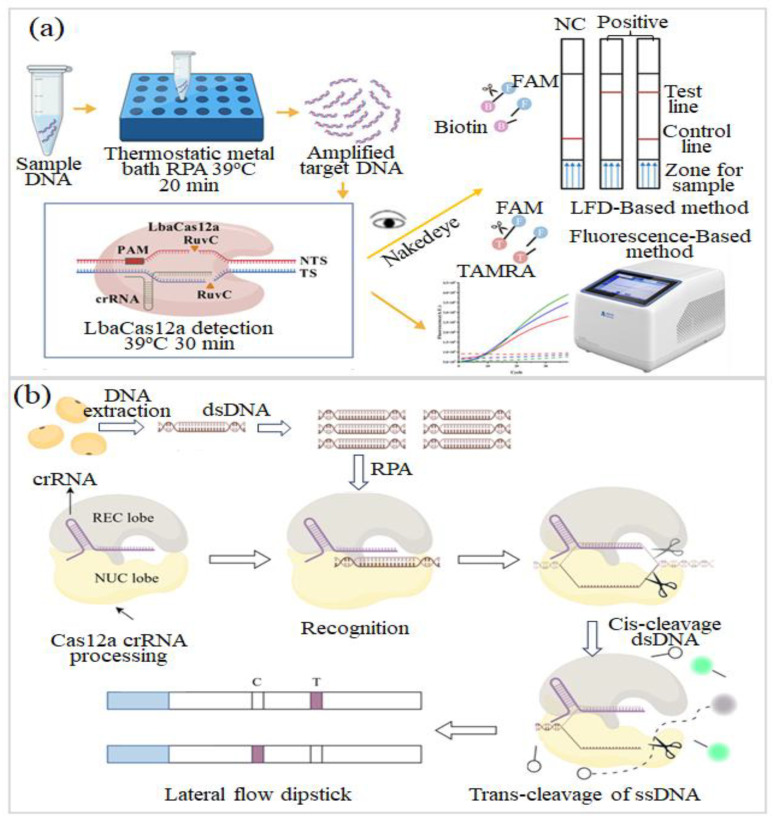
(**a**) Schematic representation of the working principle of the RPA–CRISPR/LbaCas12a–LFD system for visual detection of *Chrysotila dentata*. (Reprinted from [111]). (**b**) Schematic illustration of the RPA–CRISPR detection principle for rapid field visualization of genetically modified soybean ‘Zhonghuang 6106’. (Reprinted from [116]).

Aquaculture applications show a stronger movement toward multiplex and quantitative surveillance. Li et al. [110] developed a centrifugal microfluidic chip for shrimp pathogens using field shrimp samples and parallel real-time fluorogenic RPA for *Vibrio* spp. (V_AHPND_), white spot syndrome virus (WSSV), infectious hypodermal and hematopoietic necrosis virus (IHHNV), shrimp hemocyte iridescent virus (SHIV), and Enterocytozoon hepatopenaei (EHP). Six genetic markers were analyzed from one sample input; 5 μL reaction chambers operated at 39 °C for 20 min, with a detection limit of 10 copies/μL, a coefficient of variation below 0.10, and clinical sensitivity/specificity of 96.4%/100% compared with PCR plus sequencing. For coastal harmful algal bloom surveillance, Yu et al. [111] treated Chrysotila dentata as a field-level signal rather than a laboratory-only target. Their RPA–CRISPR/LbaCas12a–LFD strip (Figure 5a) ran at 39 °C and produced readable bands in 60 min, down to 5 × 10^−6^ pg/μL DNA or 2.82 × 10^1^ cells/mL, shortening the path beyond microscopy, qPCR, and sequencing. Fang et al. [101] further improved field quantification by embedding one-pot RPA–CRISPR/Cas12a chemistry into a closed centrifugal chip with in-run standard-curve calibration. Using shrimp, mackerel, oyster, and field shrimp samples, the system reached 6.08 copies/μL, covered 10^0^–10^4^ copies/μL, and showed qPCR-comparable performance. Collectively, these platforms move biosurveillance beyond qualitative readouts toward automated, multiplex, calibration-aware analysis; future progress depends on rugged preparation, stable reagents, matrix tolerance, and real-world validation.

### 4.2. Environmental Pathogen and One Health Biosurveillance

Environmental pathogen and One Health biosurveillance now form a common analytical ground for public health, animal production, food systems, and ecosystem protection. RPA-enabled microfluidic and biosensing platforms strengthen this ground by linking aerosol, livestock, agricultural, and antimicrobial-resistance monitoring to field-ready molecular evidence [52,107,112]. The result is not only faster detection but also earlier access to information where risk begins to emerge.

Li et al. [55] treated air as a surveillance matrix rather than a passive exposure route. Their Siam/iCASA cartridge joined portable cyclone sampling, chitosan-modified quartz-filter RNA capture, and in situ tetra-primer RPA for SARS-CoV-2 aerosols from isolation wards and public-relevant spaces. Within 25 min, the assay reached 20 copies/mL, detected RNA in 52.2% of ward aerosols, and outperformed a commercial RT-PCR kit. Feng et al. [113] applied the same aerosol-surveillance rationale to influenza subtyping, pairing one-step RT-RPA with a sealed centrifugal microfluidic chip for public-area air samples. Using only 6.25 µL, the device amplified H1N1-HA, H1N1-NA, IVB-HA, and IVB-NA within 25 min. Detection limits reached 10^2^ copies/mL for H1N1-HA and 10^1^ copies/mL for the other targets, with 100% specificity against common respiratory viruses and 96.15–100% agreement with off-chip qRT-RPA. Compared with conventional two-step RT amplification or centralized RT-PCR, the sealed chip format reduced aerosol-contamination risk while enabling subtype-level environmental screening.

At the animal-facing end of One Health surveillance, rapid outbreak control remains essential. Sun et al. [41] reported a surface-engineered μPAD for automated RPA detection of African swine fever virus (ASFV) and pseudorabies virus (PRV) in animal body fluids. Oxygen plasma/PVP treatment improved the capillary movement of viscous RPA reagents, cutting reagent-transfer time from 82 to 27 s. The resulting sample-in–answer-out device detected both ASFV and PRV at 10 copies/µL, with readouts completed in 11.5 and 14.5 min, respectively. Wu et al. [114] brought virulent-strain duck plague virus (DPV) testing closer to farm use by designing a biosensor for duck throat swabs. The assay combines RPA–CRISPR/Cas12a recognition with catalytic hairpin assembly (CHA)-assisted fluorescence amplification, reaching 0.02 fg/µL within 40 min and matching PCR-based clinical diagnoses. Such systems move veterinary diagnostics away from delayed laboratory confirmation and closer to farm-level outbreak containment.

Environmental AMR tracking is another central One Health priority. Skiba et al. [115] reported a foil-based electrochemical genosensor for unpurified asymmetric PCR amplicons of the vanB vancomycin-resistance gene, reaching below 50 nM with 100% specificity and stable microfluidic operation at 2.6–15 µL/min. Tao et al. [116] used RPA–CRISPR/Cas12a to verify the genetically modified soybean cultivar “Zhonghuang 6106” in seeds, young shoots, and blind field-like samples, with lateral-flow and fluorescence readouts available in parallel (Figure 5b). The assay reached 10 copies/µL in approximately 60 min and retained high specificity against mixed genetically modified (GM) maize, rice, cotton, rapeseed, and soybean materials. By moving GM identification into a portable assay format, this method reduces dependence on qPCR-based laboratory supervision for crop screening. Collectively, these works indicate a clear transition from instrument-heavy surveillance toward portable, matrix-aware molecular systems. The remaining challenge is no longer amplification chemistry alone but robust sampling, inhibition tolerance, quantitative calibration, reagent storage, and validation in naturally contaminated environments. Table 5 summarizes representative RPA-based environmental and One Health biosurveillance platforms, highlighting targets, assay conditions, sensing formats, and analytical performance.

### 4.3. Emerging Trends in Environmental RPA Biosurveillance

Environmental RPA diagnostics are entering a stage defined by integration, specificity, and networked surveillance. A first clear direction is the maturation of sample-to-answer platforms, where sample collection, pretreatment, nucleic acid extraction, amplification, and signal readout are housed within one compact workflow [106,110]. Such architectures reduce operator-dependent variability and make environmental testing more reproducible under field conditions. A second direction is the rapid expansion of CRISPR-assisted RPA. Cas-based recognition strengthens sequence-level specificity while remaining compatible with fluorescence, electrochemical, colorimetric, lateral-flow, and SERS readouts, thereby widening the analytical choices for complex water, food, animal, and agricultural matrices [41,100,114]. A third movement is toward autonomous and connected biosurveillance. Wireless transmission, cloud analytics, smartphone interfaces, and AI-supported interpretation may convert isolated tests into spatially resolved warning systems [108]. In the longer term, the convergence of RPA, environmental DNA analysis, One Health surveillance, and intelligent sensing could shift environmental monitoring from periodic confirmation to continuous risk tracking, supporting earlier disease control, ecosystem protection, food security, and public health preparedness.

### 4.4. Challenges and Future Perspectives in Environmental Monitoring

Environmental RPA starts from a less orderly specimen than most clinical or food-testing assays [11,106]. Targets must be recovered from open matrices—air, water, soil, plant tissue, animal fluids, and aquaculture samples—where weak nucleic-acid traces are often patchy and mixed with dispersed cells, extracellular genetic material, humic matter, salts, polysaccharides, pigments, and suspended debris. Each component may compromise nucleic-acid recovery, fluid transport, enzymatic amplification, or signal readout [12,108]. In this setting, sampling is not a preparatory step but a central part of the assay. Even highly sensitive amplification chemistry offers little practical value when sparse targets cannot first be captured, concentrated, and purified from large or variable environmental volumes.

Quantification is another unresolved issue. In environmental surveillance, a positive signal does not always translate directly into infection risk, organism viability, or outbreak severity. Residual DNA, nonviable cells, aerosol dilution, seasonal variation, and uneven spatial distribution can all complicate interpretation [107,108,111]. Field systems therefore need calibration strategies, internal controls, inhibition checks, and reference thresholds that are meaningful for each sample type. High-copy amplicons generated by RPA can generate carryover risk during outdoor or near-source testing, and closed cartridges and contamination-aware workflows are equally important.

Practical deployment also depends on field robustness. Reagents should be tolerant to temperature variations, humidity, transport, and delayed use. The device should remain practical in the field, with low power demand, simple operation, and little need for supporting equipment. For One Health and biosecurity applications, validation should extend beyond spiked samples to naturally contaminated environments, multisite testing, seasonal sampling, and agreement with accepted laboratory methods. Future environmental RPA platforms should therefore combine rugged sampling, inhibitor-tolerant preparation, stable reagent storage, quantitative or semi-quantitative readout, and secure data reporting. Such requirements are essential for moving environmental monitoring from isolated proof-of-concept assays toward reliable early-warning systems.

## 5. Intelligent, Digital, and AI-Enabled RPA Diagnostic Systems

Microfluidic and paper-based RPA systems have broadened access to molecular diagnostics, but field deployment now depends on more than amplification sensitivity. Reliable interpretation, automation, multiplexing, quantification, and real-time data handling have become central design requirements. Accordingly, RPA platforms are embedding digital sensing, automated image analysis, cloud connectivity, and machine learning to reduce operator dependence and strengthen analytical consistency. This shift is moving point-of-care testing toward intelligent diagnostic networks capable of autonomous operation, data-guided decisions, personalized care, and population-level surveillance [15,108].

### 5.1. Smartphone- and AI-Assisted RPA Diagnostics

Smartphone- and AI-assisted RPA diagnostics are reshaping molecular testing from instrument-dependent assays into portable, guided, and interpretation-ready platforms. Recent designs combine microfluidics, fluorescence or lateral-flow readout, CRISPR recognition, digital partitioning, and machine learning to improve field operation, quantitative reliability, and decentralized decision-making [117,118].

Liu et al. [15] described paper-based home nucleic acid testing as a practical route toward decentralized diagnostics. In this setting, μPADs offer inexpensive fluid handling and can be paired with intelligent signal interpretation, while broader adoption still depends on more efficient extraction, stable amplification chemistry, and standardized readout criteria. Jin et al. [50] translated Salmonella Typhimurium detection into a smartphone-assisted microfluidic RPA format with fluorescence readout. Purified bacterial DNA was used as input, and an integrated valve was used to route reagents and mix them on demand within the chip. The assay covered 1.0–1.0 × 10^5^ copies/µL and reached a detection limit of 1.0 × 10^2^ copies/µL within 30 min. By replacing 1- to 2-day culture workflows and avoiding benchtop qPCR instrumentation, this format brings rapid pathogen screening closer to field use.

**Figure 6:**
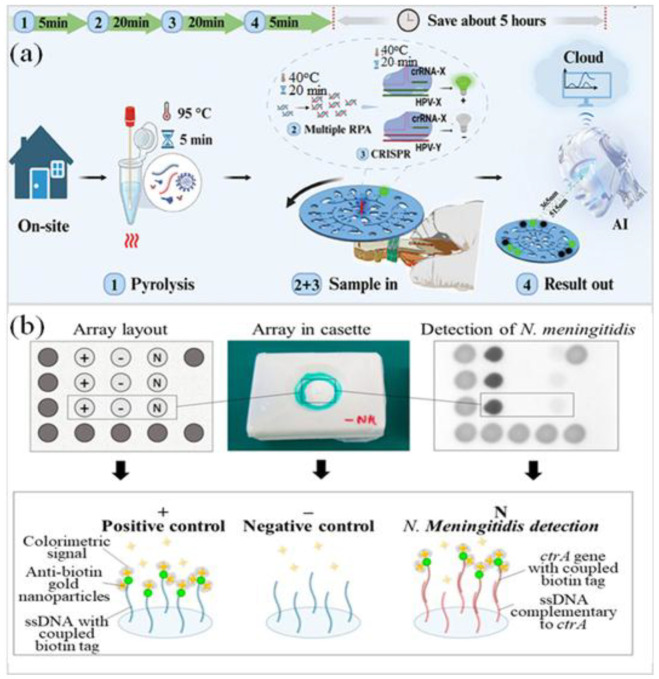
(**a**) Schematic illustration of a deep learning-enhanced hand-driven microfluidic chip and workflow for HR-HPV detection. (Reprinted from [51]). (**b**) Schematic illustration of an automated diagnostic workflow for vertical-flow microarray detection of *N. meningitidis* in low-resource settings. (Reprinted from [87]).

Smartphone-guided platforms have also opened a practical route for applying RPA to pharmacogenomic screening. Pang et al. [81] reported a sealed microfluidic wafer for HLA-B*58:01 genotyping from raw oral swab samples. The platform combines an amplification arrest mutagenesis system and locked nucleic acid technology to enhance double-stranded RPA, β-globin internal control, on-chip lateral flow readings, and smartphone-guided operation. Both targets were detected at 10 copies/μL in 25 min, with all 15 buccal swab samples giving results consistent with sequencing-based typing. For clinical screening, Xu et al. [51] presented R-CHIP, a hand-powered RPA–CRISPR microfluidic platform for high-risk HPV detection, as shown in Figure 6a. In clinical samples, the system detected HPV-16 at 10^−17^ M and HPV-18 at 10^−18^ M, delivered sample-to-result analysis within 1 h, and showed strong concordance with multiplex PCR across 300 tests. A smartphone microimaging module, supported by a ResNet-18 classifier, further enabled automated result interpretation, highlighting the value of AI-assisted readout for reducing observer bias and extending screening to community-level settings.

### 5.2. Digital, Quantitative, and Automated Microfluidic RPA Platforms

Digital, quantitative, and automated microfluidic RPA platforms are strengthening decentralized nucleic-acid testing by bringing reaction partitioning, closed-cartridge operation, programmable fluid handling, and objective readout into the same analytical workflow [42,43,119]. Digital and automated microfluidic RPA platforms address two long-standing weaknesses of conventional RPA: limited quantification and operator-dependent workflow control.

Digital and semi-digital RPA formats are strengthening quantitative molecular diagnostics. Liu et al. [120] developed a photocurable polysiloxane chamber-array chip for *E. coli* and *S. aureus* nucleic acid detection, using rapid molding and straightforward sample partitioning to cover 0–5000 copies/μL with a sensitivity of 1 copy/μL. Yang et al. [121] used light-triggered reaction control to adapt digital RPA for detecting clinical oral-pathogen DNA. Photocleavable caged primers kept amplification silent during sample partitioning and triggered the reaction only after digitization, limiting pre-amplification bias and enabling single-molecule analysis across 5–3 × 10^4^ copies/μL within 40 min. Huang et al. [122] used droplet-counting RPA–CRISPR/Cas12a for the detection of methylated FOXD3 with a detection limit of 5 copies/µL and the ability to distinguish methylation fractions as low as 0.01%. This work demonstrates the potential of a partitioned readout for RPA-based assays to detect rare biomarkers.

With automation, these systems are closer to practical deployment. Kim et al. [86] reported a disposable cartridge and automated multi-diagnostic platform for nasopharyngeal respiratory samples integrating virus capture, wash, thermal lysis, RT-RPA, and CRISPR-Cas12a fluorescence detection. The platform detected H1N1, H3N2, influenza B, and SARS-CoV-2 within 35–50 min, with on-cartridge detection limit of 1, 100, 10, and 100 copies/µL, respectively. Urrutia Iturritza et al. [87] showed how open-source robotics can streamline RPA-based detection of Neisseria meningitidis in a cerebrospinal fluid (CSF)-like matrix (as shown in Figure 6b). Magnetic-bead DNA isolation, RPA, exonuclease digestion, and paper microarray readout were assembled into a single automated sequence, bringing the total assay time down to 120 min. For user-facing diagnostics, Zhang et al. [53] presented a hand-driven spatially encoded microfluidic device integrating RPA–CRISPR/LFA chemistry with AI-assisted mobile interpretation for HPV and respiratory virus diagnostics. The workflow is compatible with cervical or vaginal swabs and respiratory specimens, supporting user-facing molecular testing outside conventional laboratory settings. The detection limit for HPV plasmids was found to be as low as 1 × 10^−18^ M, and clinical evaluation of 140 samples yielded 98.57% accuracy. The subjectivity in interpreting the results was further reduced by YOLOv8-assisted image analysis. Collectively, these platforms show that the next stage of RPA diagnostics will depend less on amplification speed alone and more on synchronized initiation, reproducible partitioning, closed automation, matrix-compatible preparation, and objective digital interpretation. Table 6 summarizes intelligent and AI-enabled RPA platforms, highlighting detection models, analytical sensitivity, automation strategy, and practical diagnostic effects.

### 5.3. Connected RPA Diagnostics and Intelligent Healthcare Systems

The next stage of RPA diagnostics will depend more on the effectiveness of assays in connecting to digital health systems than on amplification performance alone. Cloud computing, wireless communication, and data analytics are now linking microfluidic and paper-based RPA formats with wider healthcare and surveillance infrastructures [15,108]. Automatic result transfer to centralized databases can extend RPA testing from individual diagnosis to real-time outbreak monitoring, hotspot mapping, resource allocation, and public-health response. When coupled with geospatial analysis, these connected workflows could further strengthen population-level tracking during infectious disease events.

At the technical level, the convergence of AI, digital microfluidics, CRISPR recognition, quantitative readout, and cloud connectivity is turning RPA systems into intelligent diagnostic platforms rather than isolated analytical tools [51,53,108,117]. Future formats may combine automated interpretation, predictive analytics, and clinical decision support in portable or home-based devices. Such development could shift molecular diagnostics from centralized laboratories toward patient-centered, digitally connected care while preserving professional oversight and data-driven surveillance capacity.

### 5.4. Challenges and Future Perspectives in Intelligent RPA Diagnostic Systems

Intelligent, digital, and AI-enabled RPA systems introduce new opportunities, but they also add technical and regulatory demands beyond amplification chemistry [15,108]. Image-based interpretation depends strongly on lighting conditions, camera optics, focal distance, background color, chip alignment, reagent lot, and sample matrix. A model trained under one device setting may not perform equally well across different smartphones, readers, cartridges, or field environments [117,118]. AI-assisted diagnostics gain clinical or field value only when their predictions are backed by solid evidence. This requires representative training data, clearly reported validation, independent testing, and preset criteria for borderline or weak signals.

Digital and automated RPA platforms bring their own engineering burden. Quantitative readout is only as reliable as the partitioning: droplets or chambers must fill evenly, remain sealed, start reactions at nearly the same time, and support defensible calls between positive and negative partitions, especially near the low-copy limit [42,43,119]. Small imperfections—evaporation, leakage, partial filling, spurious amplification, or local thermal drift—can quickly bias the count. Automation removes much of the handwork but adds new places for error: stuck valves, delayed reagent release, bubbles, clogged channels, image-reading mistakes, and software-driven misclassification. For use beyond trained laboratories, internal controls and automatic quality gates should be treated as core design requirements, not optional safeguards.

Connected diagnostics need evaluation beyond the chemistry of the assay. When cloud reporting, wireless transfer, geotagged results, and AI-based interpretation become part of the workflow, the test also inherits concerns over privacy, cybersecurity, audit records, software updates, interoperability, and regulatory review. For clinical translation, validation should span the full operating chain, from biochemical reaction and cartridge performance to algorithm behavior, user interface, data transfer, and final decision logic. Analytical performance alone will not secure adoption. Smartphone readout can bring molecular testing closer to clinics, homes, farms, processing plants, and surveillance sites, but routine use will hinge on the real cost of cartridges, readers, cloud support, and maintenance. The next stage should focus less on connectivity as a feature and more on usable infrastructure: shared benchmark datasets, interpretable outputs, consistent imaging, built-in quality checks, secure data handling, and regulatory routes suited to software-supported molecular diagnostics.

## 6. Conclusions and Future Outlook

Microfluidic and paper-based RPA technologies have moved isothermal amplification from a bench-level reaction into deployable molecular testing workflows for biomedical diagnosis, food-safety inspection, environmental monitoring, and public-health surveillance. Microfluidic formats provide precise fluid control, reduced reagent consumption, faster reaction handling, and compact operation. Paper-based platforms bring low-cost fabrication, capillary-driven fluid transport, and minimal instrument requirements, making molecular testing more accessible in low-resource and point-of-need settings [11,12,15,17,123]. Across the studies reviewed here, RPA serves not simply as a rapid amplification reaction but as the central engine that coordinates extraction, target recognition, signal generation, and user-facing diagnostic readout.

A major development in this field is the functional coupling of RPA with technologies that improve specificity, quantification, readout, and interpretation. CRISPR recognition strengthens sequence discrimination; digital partitioning supports molecular counting; electrochemical interfaces enable compact quantitative transduction; and smartphone-based imaging improves user-facing readout [31,52,108,124,125]. These combinations have broadened the application space of RPA from pathogen screening to pharmacogenomic testing, cancer-associated biomarker analysis, antimicrobial resistance monitoring, foodborne surveillance, and environmental biosensing [52,76,80,81,82]. The strongest platforms are those in which amplification, sample handling, signal generation, and result interpretation are designed as one workflow rather than assembled as separate steps.

Several barriers still limit routine deployment. Sample preparation remains the most persistent bottleneck, especially for clinical specimens, food matrices, and environmental samples that contain inhibitors, viscous components, low target abundance, or variable background signals [14,126]. Cartridge-based formats that combine extraction, purification, amplification, and readout have begun to address these issues, but stronger robustness across sample types is still needed [87,127]. Multiplex RPA involves more than expanding the primer pool. Reliable performance depends on a careful balance among primer architecture, reaction partitioning, and signal discrimination, since primer competition, biased amplification, and cross-reactivity can readily compromise assay specificity [24,66,70]. Quantification remains equally difficult. Digital RPA and droplet-based strategies improve molecular counting by dividing reactions into smaller compartments, but practical deployment still requires simpler fabrication, lower analytical burden, and workflows that can operate reliably outside highly specialized laboratories [42,43,47,49,52,53].

Translation also depends on manufacturing maturity, regulatory readiness, and a realistic cost structure. Reagent stability is a central issue because RPA enzymes, primers, probes, CRISPR reagents, and reporter chemistries must remain active during storage, shipping, and use outside temperature-controlled laboratories [17,18,108,128]. Lyophilization, humidity protection, sealed reagent compartments, and compatibility between dried reagents and device materials therefore require careful optimization rather than late-stage adjustment. Manufacturing consistency presents a separate challenge. Paper devices are shaped as much by the substrate as by the assay chemistry. Fiber uniformity, wicking rate, surface modification, and reagent placement determine whether liquids reach the reaction zone in a predictable manner. Microfluidic cartridges impose their own manufacturing tolerances. Channel dimensions, sealing strength, valve actuation, chamber volume, and optical or electrochemical coupling must be reproduced from lot to lot; modest deviations can delay amplification, dampen signal readout, or erode quantitative concordance. Quality assessment should therefore extend beyond amplification performance to the device itself, covering lot-to-lot verification, built-in controls, contamination checks, shelf-life testing, user-error analysis, and concordance with accepted reference methods. Regulatory review becomes more demanding once these systems move from research screening toward clinical diagnosis. Analytical sensitivity, specificity, matrix tolerance, reproducibility, stability, usability, and risk management must be demonstrated with well-defined validation protocols. Cost will also shape adoption. A technically elegant cartridge may have limited field value if fabrication, reagent packaging, reader hardware, cold-chain logistics, or per-test pricing remain too high for community clinics, farms, food-processing sites, or environmental surveillance programs. For this reason, practical translation should be considered from the beginning of platform design, with performance, manufacturability, quality assurance, regulatory documentation, and affordability developed as connected requirements rather than separate downstream tasks.

Connected analytical tools will further shape how RPA platforms are used. Machine-learning-assisted image analysis, smartphone interfaces, cloud reporting, and automated quality-control gates can reduce subjective interpretation and support structured data transfer into clinical or surveillance networks [15,51,52,53,54,108]. In parallel, the One Health framework places human diagnostics, veterinary testing, food safety, agricultural monitoring, and environmental surveillance within a shared molecular-warning landscape [52,108]. RPA-based platforms are well suited to this direction because the same amplification principle can be adapted across clinics, farms, food chains, and field sampling sites.

Beyond the application areas emphasized in this review, veterinary outbreak control, antimicrobial-resistance surveillance, forensic identification, plant protection, and biosecurity-oriented monitoring may benefit from the same platform principles: stable reagents, matrix-aware preparation, closed amplification, objective readout, and data-ready reporting. These directions should be evaluated with the same standards applied to clinical and food-safety systems, particularly reproducibility, field robustness, user safety, and agreement with accepted reference methods.

Microfluidic and paper-based RPA technologies have matured into versatile platforms for accessible molecular diagnostics across clinical, food-safety, environmental, and biosurveillance settings. Although challenges related to sample preparation, quantification, standardization, and commercialization remain, ongoing advances in CRISPR diagnostics, digital microfluidics, artificial intelligence, and connected healthcare infrastructures are rapidly accelerating technological maturation. Together, CRISPR-guided recognition, digital microfluidics, electrochemical sensing, smartphone readout, and AI-assisted analysis are pushing RPA diagnostics beyond single-function assays. Their combined use is opening practical routes for deployment in healthcare, precision medicine, food safety, environmental surveillance, and biosurveillance. The next priority is to translate this technological convergence into reproducible devices, validated workflows, and implementation pathways that can be evaluated across clinical, food-safety, and environmental settings.

## Figures and Tables

**Table 1:** Comparison of RPA and PCR for biomedical molecular diagnostics.

Category	RPA	PCR
**Principle**	Isothermal amplification using recombinase, primers, SSB proteins, and strand-displacing polymerase.	Thermal cycling amplification through denaturation, annealing, and extension.
**Temperature**	Mild, nearly constant temperature, usually 37–42 °C.	Requires repeated cycling, typically 90–95 °C, 50–70 °C, and 70–75 °C.
**Assay time**	Rapid; often completed within 10–30 min.	Slower in conventional formats; micro-PCR can shorten cycling time.
**Instrumentation**	Simple heaters or portable readers are usually sufficient.	Requires precise thermal cycling and stable temperature control.
**Biomedical use**	Suitable for point-of-care testing, emergency screening, field diagnosis, and low-resource settings.	Suitable for confirmatory diagnosis, viral-load monitoring, genotyping, and regulated clinical testing.
**Advantages**	Fast, low-power, portable, and compatible with paper-based or microfluidic devices.	Reliable, highly validated, quantitative, specific, and broadly accepted in clinical laboratories.
**Limitations**	Quantification remains less mature; primer design, non-specific amplification, and contamination require careful control.	Instrument-dependent, less portable, and less convenient for decentralized testing.
**Best scenario**	Rapid screening when speed, simplicity, and accessibility are priorities.	Laboratory confirmation when accuracy, standardization, and traceability are essential.
**Overall role**	Practical amplification chemistry for decentralized molecular diagnostics.	Benchmark method for high-confidence biomedical nucleic acid testing.

**Table 2:** Representative paper-based RPA devices summarized by substrate type, target analyte, detection strategy, assay time, limit of detection, and practical advantages.

Ref.	Substrate Type/Device Format	Target Analyte	Detection Strategy	Assay Time	LOD	Practical Advantages
[32]	Origami microfluidic device	*Salmonella enterica*	Dipstick extraction, RPA, and lateral-flow visualization	20 min	260 CFU/mL; 58 CFU/mL after 6 h enrichment	Integrates extraction, amplification, and visual readout in a foldable PES format with enhanced RPA compatibility.
[35]	μPAD	*Salmonella Typhimurium*	RPA–Cas12a with SERS readout	~45 min	4.04 CFU/mL	Combines paper fluidics, CRISPR specificity, and Raman enhancement for sensitive food-matrix detection.
[36]	μPAD with lyophilized reagents	HPV16 E7 dsDNA	RPA–Cas12a, colorimetric readout	60 min	100 pM	Enables instrument-free visual screening, with dried reagents supporting portable, storage-stable deployment.
[37]	Nucleic-acid lateral-flow assay	SARS-CoV-2 RdRp RNA	RT-RPA with lateral-flow readout	30 min	4.1 copies/µL	Delivers rapid strip-based visual detection without electrophoresis or fluorescence instrumentation.
[38]	Hand-warmer-heated paper device	*Neisseria gonorrhoeae*	RPA, lateral-flow readout	30–35 min	10 DNA copies or 1 CFU/mL	Uses low-cost heating and paper processing for field-ready STI testing in resource-limited settings.
[39]	Lyophilized paper-based platform	Norovirus RNA	RT-RPA with fluorescence readout	45 min	1 copy/µL	Retains room-temperature activity and enables testing across water, lettuce, and oyster matrices.
[40]	Foldable μPAD	*Vibrio parahaemolyticus*	RPA reagents, fluorescence imaging	20 min	10^2^ CFU/mL	Improves on-paper reaction mixing, reduces equipment demand, and supports rapid seafood and seawater screening.
[41]	Surface-engineered μPAD	African swine fever virus	RPA with fluorescence readout	14.5 min for PRV	10 copies/µL	Accelerates viscous RPA flow via plasma/PVP treatment, enabling rapid veterinary outbreak screening.
[42]	Wax-gated RPA-PAD	*Plasmodium falciparum*	RPA with scanner or smartphone image analysis	~35 min	28 parasites/mL	Enables low-resource malaria quantification with inexpensive paper handling and image-assisted interpretation.
[43]	Valve-integrated μPAD	HPV16 E7 gene	RPA–CRISPR/Cas12a fluorescence detection	35 min	1 fM	Combines fluidic control, amplification, and CRISPR readout for instrument-light cervical cancer screening.

LOD: Limit of detection; PES: Polyethersulfone; STI: Sexually transmitted infection.

**Table 3:** Overview of representative RPA-based biomedical diagnostic platforms by target, amplification conditions/time, sensing chemistry, readout mode, and analytical performance.

Ref. and Years	Detection Target	RPA Condition/Time	Active Substance/Sensing Element	Detection Mode	Detection Range and LOD
Kyung et al., 2024 [37]	SARS-CoV-2 RdRp RNA	Two-step RT-RPA: reverse transcription at 42 °C for 15 min, followed by RPA at 38 °C for 15 min	Tailed RT-RPA primers, AuNP–reporter probe	Colorimetric readout	R: 400 to 0.01 copies/µL; LOD: 4.1 copies/µL
Huang et al., 2021 [58]	SARS-CoV-2 and measles virus RNA	First-stage RPA at 37 °C for 10 min, followed by second-stage LAMP at 65 °C for 50 min	RPA primers, LAMP primers, fluorescence readout reagent	Fluorescence detection	R: 10^4^ to 10^1^ copies; LOD: 10 copies. 40 nasopharyngeal samples
Chen et al., 2025 [61]	Monkeypox virus DNA	Single-step RPA–CRISPR/Cas13a at 37 °C for 30 min	GO@Pt 2D nanozyme, FAM–ssRNA–biotin reporter	Colorimetric CRISPR assay	R: - LOD: 1 copy/µL 40 clinical samples
Wang et al., 2026 [62]	HPV16 and HPV18 plasmids	RPA at 37 °C for 20 min, followed by Cas12a reaction at 37 °C for 20 min	Lyophilized RPA mix, Cas12a/crRNA complex	Lateral-flow visual readout	R: - LOD: 20 copies/reaction
Chen et al., 2025 [63]	*Mycoplasma hominis* 16S rRNA	RPA at 37 °C for 20 min, followed by CRISPR/Cas12a at 37 °C for 30 min	Cas12a/crRNA, bio-dig reporter probe	Visual and grayscale readout	R: 10^5^ to 1 copies/µL LOD: 2 copies/reaction 18 clinical samples
Hua et al., 2026 [64]	SARS-CoV-2 ssRNA	RT-aRPA (asymmetric), 42 °C; 10 min; sample-to-result ~20 min	DRCP-based ECNA sensing layer	Electrochemical ECNA biosensor	R: 30 pM–20 nM; LOD: 12.4 pM
Ma et al., 2025 [70]	6 viruses: FLUAV, FLUBV, HPIV-1, HPIV-2, HPIV-3, and SARS-CoV-2	FARPA-chip at 42 °C for 20 min, followed by 95 °C for 1 min and 60 °C for 10 min	Universal RPA primers, FEN1 invasive reaction probes	Multiplex fluorescence detection	Tested at 5, 10, 50, and 200 RNA copies/target; LOD: 10 copies of each target
Wang et al., 2026 [71]	*Staphylococcus aureus*, *Escherichia coli*, and *Listeria monocytogenes*	Bacterial capture/RPA at 37 °C for 15 min; Cas12a reaction at 37 °C for 40 min; enzyme stopped at 60 °C for 5 min	MXene-Fe@Apt nanosheets, bacterial aptamers, released activator DNA, RPA reagents	Fluorescence assay	R: 50–10^6^ CFU/mL for *S. aureus*; 50–10^6^ CFU/mL for *E. coli*; 10^2^–10^6^ CFU/mL for *L. monocytogenes*. LODs: 29, 16, and 76 CFU/mL
Chen et al., 2026 [79]	HFMD-relevant enteroviruses EV-A71, CV-A16, CV-A6, CV-A10	Integrated one-pot RT-RPA/CRISPR maintained at ~ 39 °C during a 1 h	Cas12a/crRNA + FAM-BHQ ssDNA reporter	Fluorescence	Evaluated at 10 aM, 100 aM, 1 fM; LOD: 10 aM

DRCP: Dual recognition sequences-containing capture probe; FARPA: Fen1-aided RPA; HFMD: Hand, foot, and mouth disease; ECNA: Electrochemical nucleic acid.

**Table 4:** Representative RPA-based food safety diagnostic platforms summarized by detection target, amplification condition/time, sensing element, readout mode, dynamic range, and limit of detection.

Ref. and Years	Detection Target	RPA Condition/Time	Active Substance/Sensing Element	Detection Mode	Detection Range and LOD
Zhuang et al., 2022 [35]	*Salmonella typhimurium*	RPA-Cas12a-μPAD about 45 min	CRISPR/Cas12a, crRNA, linker ssDNA	Raman spectrometric readout	R: 1–10^8^ CFU/mL; LOD: 3.72 CFU/mL in milk and 4.04 CFU/mL in meat
Zhou et al., 2025 [90]	*Shigella*; *ipaH* gene	RPA optimized at 39 °C for 25 min; SDA at 58 °C for 10 min	G4/Nt.BstNBI-engineered RPA primer; Bst 2.0 polymerase	Visual colorimetric readout	Tube assay LOD 3 × 10^−3^ ng/µL; on-chip LOD 3.5 × 10^−4^ ng/µL
Nguyen et al., 2025 [91]	*Salmonella Typhimurium*; *invA* gene	RPA at 37 °C for 50 min; full process 75 min	Filter-paper DNA capture; freeze-dried RPA powder	Capillary electrophoresis	R: 10^2^–10^7^ CFU/mL; LOD 10^3^ CFU/mL
Guo et al., 2024 [93]	*Staphylococcus aureus*	RPA: 30 min; CRISPR/Cas12a cleavage on electrode: 37 °C for 30 min	CRISPR/Cas12a, target-specific crRNA, MB-labeled hairpin DNA	Electrochemical biosensor	Pure culture: 1.04 × 10^1^–1.04 × 10^8^ CFU/mL, LOD 3 CFU/mL; milk: 1.07 × 10^1^–1.07 × 10^7^ CFU/mL
Chen et al., 2025 [95]	*Salmonella*	RPA at 41 °C for 90 min; eLFS visual readout 10 min	CRISPR/Cas12a, crRNA, FITC-ssDNA-Bio probe	Electrochemical lateral flow strip	R: 3.84–3.84 × 10^7^ CFU/mL; LOD 1.96 CFU/mL
Guo et al., 2025 [96]	*Staphylococcus aureus* and *Salmonella*	RPA was performed at 37 °C for 30 min	CRISPR/Cas12a, target-specific crRNA, Fc-labeled ssDNA probes	Electrochemical biosensor	*S. aureus*: 1.06 × 10^1^–1.06 × 10^7^ CFU/mL, LOD 3 CFU/mL; *Salmonella*: 1.04 × 10^1^–1.04 × 10^7^ CFU/mL, LOD 3 CFU/mL
Xu et al., 2025 [100]	*Vibrio parahaemolyticus*	RPA: 37 °C for 30 min, followed by 80 °C for 10 min protein inactivation	CRISPR/Cas12a, tdh-/trh-specific crRNA	Electrochemical biosensor	R:10^1^–10^6^ CFU/mL; LOD 10.6 CFU/mL
Hanze et al., 2023 [104]	*DNA from toxic microalgae*	RPA using TwistAmp Basic Kit; 37 °C for 30 min	Thiolated DNA capture probes forming SAMs, HRP-tagged reporter probe	Electrochemical biosensor	1 pM synthetic DNA about 1500 microalgal cells/reaction
Dong et al., 2023 [105]	Six pathogen targets	Total 50 min at 37 ± 1 °C	CRISPR/Cas13a, target-specific crRNA	Electrochemical/optical CRISPR biosensor	LOD down to 30 zM within 45 min

Six pathogen targets (*Yersinia pestis*, *Francisella tularensis*, *Chlamydia psittaci*, *Burkholderia mallei*, *Burkholderia pseudomallei*, and *Brucella melitensis*).

**Table 5:** Representative RPA-based platforms for environmental monitoring and One Health biosurveillance, summarized by detection target, amplification condition/time, sensing element, readout mode, analytical range, and detection limit.

Ref. and Years	Detection Target	RPA Condition/Time	Active Substance/Sensing Element	Detection Mode	Detection Range and LOD
Sun et al., 2026 [41]	*ASFV* and *PRV*	RPA at 42 °C; optimized readout times: 11.5 min	RPA reagents, heating module, fluorescence probe	Fluorescence detection	R: 0–10^4^ copies/µL; LOD: 10 copies/µL
Ji et al., 2025 [52]	*Influenza A virus* and *influenza B virus*	RPA on AM-DMF chip at 39 °C for 25 min	AI droplet navigation, RPA primers, and exo-probes	Fluorescence detection	R: 10^1^–10^4^ copies/µL; LoD: 6.03 copies/µL
Xu et al., 2025 [100]	*Vibrio parahaemolyticus* in aquatic foods	RPA at 37 °C for 30 min, followed by 80 °C for 10 min protein inactivation	CRISPR/Cas12a, tdh-/trh-specific crRNA	Electrochemical biosensor	R: 10^1^–10^6^ CFU/mL; LOD: 10.6 CFU/mL
Yang et al., 2022 [106]	*Ustilaginoidea virens*	RPA-LFD performed at 37 °C for 20 min	RPA primers/probe, lateral flow dipstick	Visual test/control-line readout	R: 10^2^–10^5^ CFU/mL LOD: 10^2^ CFU/mL
Sfragano et al., 2024 [107]	*Sulfonamide-resistance* genes ***sul1*** and ***sul4*** from *E. coli*	RPA at 37 °C for 20 min	Capture probe/signaling probe sandwich	Electrochemical biosensor	R: 0.1–10 nmol/L; LOD: 44.2 pmol/L
Sun et al., 2025 [109]	*Bursaphelenchus xylophilus*	Boiling lysis at 95 °C for 10 min; RPA at 39 °C for 15 min	Portable microfluidic system, boiling lysis unit	Immunochromatographic strip readout	Positive strip results within ~40 min
Li et al., 2024 [110]	V_AHPND_, WSSV, IHHNV, SHIV, and EHP	On-chip RPA in 5 μL chambers at 39 °C for 20 min	Pre-immobilized RPA primers, fluorogenic exo probes	Centrifugal microfluidic multiplex detection	R: 1–10^6^ copies/μL; LOD:10 copies/μL
Xu et al., 2024 [112]	*Phytophthora cinnamomi* genomic	RPA at 37 °C for 10 min	CRISPR/Cas12a, Pcinn204169-specific RPA primers	Fluorescence detection	R: 10 ng to 10 fg; LOD: 10 pg genomic DNA
Feng et al., 2025 [113]	*Influenza A H1N1* and *Influenza B virus*	Amplification at 42 °C and completed within 25 min	Preloaded target-specific primers and fluorescent probes	Fluorescence detection	R: 10^6^–10^0^ copies/mL; LOD: 10^2^ copies/mL
Wu et al., 2026 [114]	*Virulent duck plague virus strain*	RPA–CRISPR/Cas12a reaction at 37 °C for 30 min	RPA primers, CRISPR/Cas12a–crRNA recognition	Fluorescence detection	R: 10^−1^–10^5^ fg/µL; LOD: 0.02 fg/µL
Tao et al., 2025 [116]	GM soybean “Zhonghuang 6106”	RPA optimized at 39 °C for 20 min	CRISPR/Cas12a, event-specific RPA primers, crRNA	Visual lateral-flow dipstick	R: 1–20,000 copies/µL; LOD 10 copies/µL

AM-DMF: Active-matrix digital microfluidic; ASFV: African swine fever virus; EHP: *Enterocytozoon hepatopenaei*; IHHNV: Infectious hypodermal and hematopoietic necrosis virus; SHIV: Shrimp hemocyte iridescent virus; WSSV: White spot syndrome virus.

**Table 6:** Representative intelligent, digital, smartphone-assisted, and AI-enabled RPA diagnostic platforms for decentralized molecular testing.

Ref. and Years	Detection Model	Target and LOD	RPA Application Method/Time	Uses and Effects
Siriyod et al. (2025) [42]	Image-assisted RPA-PAD with wax-gate control	*Plasmodium falciparum*; LOD: 28 parasites/mL by scanner, 46 parasites/mL by smartphone	Smartphone/scanner-assisted solid-phase RPA-PAD; ~35 min	✧ Enhances low-resource malaria diagnostic access ✧ Supports visual and smartphone readout ✧ Reduces lateral-flow strip cost burden
Liu et al. (2025) [43]	Valve-integrated μPAD fluorescence RPA–CRISPR platform	HPV16 E7 gene; LOD: 1 fM with circular reporter	Intelligent μPAD RPA–CRISPR/Cas12a; 35 min	✧ Enables sensitive cervical cancer screening ✧ Integrates amplification and CRISPR detection ✧ Reduces dependence on bulky instruments
Jin et al. (2023) [50]	Smartphone-assisted real-time fluorescence microfluidic biosensor	*Salmonella typhimurium* DNA; LOD: 1.0 × 10^2^ copies/µL	Intelligent microfluidic RPA with versatile valve; 30 min	✧ Supports on-site *Salmonella* screening workflows ✧ Improves mixing and flow control ✧ Reduces dependence on benchtop qPCR
Xu et al. (2025) [51]	AI-enabled hand-driven R-CHIP platform	HR-HPV; LOD: 10^−17^ M for HPV-16, 10^−18^ M for HPV-18	AI-enabled RPA–CRISPR with ResNet-18 readout; <1 h	✧ Supports community-level HR-HPV screening programs ✧ Automates mobile fluorescence image classification ✧ Shortens PCR-centered diagnostic workflows substantially
Zhang et al. (2025) [53]	AI-enhanced MACRO home molecular testing system	HPV subtypes, SARS-CoV-2, influenza A/B; attomolar sensitivity	AI-enabled spatial-encoding RPA–CRISPR/LFA; ≤60 min	✧ Enables privacy-preserving home molecular testing ✧ Reduces extraction and heating requirements ✧ Enables AI-guided result interpretation
Pang et al. (2026) [81]	Smartphone-controlled centrifugal microfluidic LFA platform	HLA-B*58:01 and β-globin; LOD: 10 copies/µL	Intelligent LNA-enhanced duplex RPA; <25 min	✧ Supports pharmacogenomic allopurinol risk screening ✧ Combines genotyping with internal control ✧ Avoids bulky fluorescence optical modules
Urrutia Iturritza et al. (2024) [87]	Open-source robotic automated diagnostic workflow	*Neisseria meningitidis* ctrA in CSF matrix; formal LOD: NR	Automated robotic RPA workflow with paper microarray; <2 h	✧ Reduces manual pipetting variability risks ✧ Increases accessible modular workflow automation ✧ Supports low-resource infectious disease testing
Nouwairi et al. (2025) [119]	Programmable microfluidic real-time amplification–HRM instrument	GAPDH/HeLa gDNA; formal LOD: NR; dilution series to 0.001–10 ng/µL	Programmable microfluidic RPA plus HRM; 20 min + <4 min	✧ Identifies false-positive RPA signals ✧ Avoids electrophoresis or sequencing confirmation ✧ Supports rapid point-of-need genomic analysis

CSF: Cerebrospinal fluid; LFA: Lateral flow assay; LNA: Locked nucleic acid; MACRO: Artificial intelligence-enhanced hand-driven microfluidic system; PAD: Paper-based analytical device.

## Data Availability

No new data were created or analyzed in this study.
